# Role of Mn^2+^ and Compatible Solutes in the Radiation Resistance of Thermophilic Bacteria and Archaea

**DOI:** 10.1155/2012/845756

**Published:** 2012-11-14

**Authors:** Kimberly M. Webb, Jocelyne DiRuggiero

**Affiliations:** Department of Biology, Johns Hopkins University, Baltimore, MD 21218, USA

## Abstract

Radiation-resistant bacteria have garnered a great deal of attention from scientists seeking to expose the mechanisms underlying their incredible survival abilities. Recent analyses showed that the resistance to ionizing radiation (IR) in the archaeon *Halobacterium salinarum* is dependent upon Mn-antioxidant complexes responsible for the scavenging of reactive oxygen species (ROS) generated by radiation. Here we examined the role of the compatible solutes trehalose, mannosylglycerate, and *di-myo-inositol* phosphate in the radiation resistance of aerobic and anaerobic thermophiles. We found that the IR resistance of the thermophilic bacteria *Rubrobacter xylanophilus* and *Rubrobacter radiotolerans* was highly correlated to the accumulation of high intracellular concentration of trehalose in association with Mn, supporting the model of Mn^2+^-dependent ROS scavenging in the aerobes. In contrast, the hyperthermophilic archaea *Thermococcus gammatolerans* and *Pyrococcus furiosus* did not contain significant amounts of intracellular Mn, and we found no significant antioxidant activity from mannosylglycerate and di-*myo*-inositol phosphate *in vitro*. We therefore propose that the low levels of IR-generated ROS under anaerobic conditions combined with highly constitutively expressed detoxification systems in these anaerobes are key to their radiation resistance and circumvent the need for the accumulation of Mn-antioxidant complexes in the cell.

## 1. Introduction

Ionizing radiation (IR) is of particular interest in biology because its exposure results in severe oxidative stress to all the cell's macromolecules. The vast majority of cellular insults under aqueous conditions are caused by indirect effects, through the action of reactive oxygen species (ROS) formed by the radiolysis of water and generating hydroxyl radicals (HO^•^), superoxide (O_2_^•−^) and hydrogen peroxide (H_2_O_2_) [1]. DNA-associated water molecules that undergo radiolysis become an immediate threat for nucleic acids, generating oxidized DNA bases and sugar moieties, abasic sites, strand breaks, and cross-links to proteins [1, 2]. Proteins are attacked by ROS introducing carbonyl residues, amino acid radical chain reactions, cross-linking, and ultimately resulting in proteins inactivation and denaturation [3, 4]. Proteins with [4Fe–4S] clusters are particularly susceptible to O_2_^•−^ and H_2_O_2_ attack, resulting in the release of ferrous ion and the formation of HO^•^ via the Fenton reaction [5]. Prevention of ROS-mediated cellular damage is therefore key for surviving IR exposure.

While it was thought that DNA lesions, and in particular DNA double-strand breaks (DSBs), were the most cytotoxic lesions resulting from IR exposure, recent findings regarding the repair of DNA from IR damage and the fact that IR-sensitive and IR-resistant organisms suffer the same number of DNA DSBs for an equivalent dose of IR (~0.01 DSB/Gy/Mbp) strongly departed from this dogma [6, 7]. It is now established that proteins are major targets of radiation damage and that protection against protein oxidation is an essential process for survival from IR exposure [8, 9].

Regarding the mechanisms underlying IR resistance, recent studies with the halophilic archaeon *Halobacterium salinarum *revealed the critical role played by nonenzymatic antioxidant processes in the radioresistance of this organism [10, 11]. *H. salinarum*, in addition to being adapted to high salt, also exhibits high-level resistance to desiccation, high pressure, UV radiation, and IR [11–14]. Its *D*_10_, the dose of radiation in Gray (Gy) that reduces the survival of a population by 90%, is 5 kGy [13]. Measurements of *H. salinarum *cell interior revealed a high manganese/iron (Mn/Fe) ratio similar to that of the extremely radiation-resistant bacterium *Deinococcus radiodurans* (*D*_10_ 12 kGy) and other IR resistant microorganisms [9, 10]. Further work with *D. radiodurans* elegantly established the key role played by Mn^2+^-peptide complexes in this bacterium's radiation resistance [15] and in yeast, *in vivo* studies showed the important function of Mn-orthophosphate complexes in oxidative stress [16]. In *Bacillus*, Mn^2+^-dipicolinic complexes are implicated in the stress resistance phenotypes of spores, including IR, wet and dry heat [17], and cyanobacteria, which are extremely resistant to IR and desiccation, accumulate Mn^2+^ and mycosporine-like amino acids [18]. In *H. salinarum* enzyme-free cell extracts rich in Mn, phosphate, amino acids, and peptides provided a high level of enzyme protection, *in vitro*, against the deleterious effect of IR, underlying that the critical role of Mn antioxidant complexes in radiation resistance also extends to archaea [11]. Cellular accumulation of Mn together with a variety of organic and inorganic ligands may be a widespread mechanism to surviving oxidative stress, and there is evidence that this may also extend to simple animals such as rotifers [19]. 

Many extremophiles have been found to be resistant to IR, suggesting that radiation resistance is a fortuitous consequence of a high tolerance to other environmental stressors [20]. From studies with *D. radiodurans* and environmental isolates, a strong link was established between desiccation and IR resistance [21, 22]. Both types of stresses generate ROS and inflict severe oxidative damage to all the macromolecules of the cell [23]. However, no direct correlation was found between desiccation tolerance and radiation resistance among (hyper)thermophilic archaea [20, 24]. The distribution of extremely IR-resistant organisms in the phylogenetic tree of life is not limited to prokaryotes. Recent work has revealed the high level of IR resistance of several eukaryotes including the basidiomycete fungus *Ustilago maydis *[25], the freshwater invertebrate animal *Philodina roseola *[26], the water bear* Milnesium tardigradum *[27], and the roundworm *Caenorhabditis elegans *[28]. A number of thermophilic archaea and bacteria have also been found to be IR resistant, including the sulfate-reducing *Archaeoglobus fulgidus*, methanogens such as *Methanocaldococcus jannaschii*, the hyperthermophiles *P. furiosus, Thermococcus radiotolerans,* and *Thermococcus gammatolerans* [20, 29–31], and the thermophilic bacteria *Rubrobacter xylanophilus *and* Rubrobacter radiotolerans* [32, 33]. However, while IR-resistant organisms are distributed across the three domains of life, this distribution can vary dramatically between organisms of the same family and even between species [7].

Thermophilic bacteria and archaea inhabit diverse environments and can survive multiple stresses including desiccation, radiation, pressure, and pH extremes together with high temperature [20, 34, 35]. Thermophiles are distinguished by their ability to grow at or above temperatures exceeding 50°C [36], which demand that their macromolecules resist not only the thermal denaturing effects of heat, but also the attendant burden of elevated oxidative stress arising from metabolic processes. Many thermophiles are also halotolerant [37, 38], and collectively, these organisms are characterized by the accumulation of amino acids, sugars, polyols, and derivatives thereof (compatible solutes) [39]. Compatible solute accumulation is conventionally attributed to protecting cells from osmotic stress and heat shock and has been shown to stabilize proteins *in vitro* [38, 40]. Mannosylglycerate (MG) is widely distributed among thermophiles, and the cellular concentration of MG has been shown to increase in response to salt stress [38]. Di-*myo*-inositol phosphate (DIP), a compatible solute exclusively found in thermophiles, is accumulated in the cell in response to thermal stress [38, 41]. Both MG and DIP have been studied for their ability to protect proteins *in vitro* against thermal stress and freeze drying [42–45]. 

In this study, we investigated the role played by the compatible solutes found in two IR-resistant thermophilic bacteria, *R. xylanophilus* (*D*_10_ 6 kGy) and *R. radiotolerans *(*D*_10_ 10 kGy), and two IR-resistant hyperthermophilic archaea, *P. furiosus *(*D*_10_ 3 kGy) and *T. gammatolerans *(*D*_10_ 6 kGy). We showed that under aerobic conditions, compatible solutes accumulated by thermophilic bacteria confer IR resistance to enzymes *in vitro* and that radioprotection is mitigated by the presence of both trehalose and  Mn^2+^. With regard to hyperthermophilic archaea, the anaerobic environment contributes to their IR resistance, which was the most significant factor for protection of enzymes *in vitro*. 

## 2. Materials and Methods

### 2.1. Growth Conditions

Rubrobacter* radiotolerans* (DSM 5868) and *Thermococcus gammatolerans *(DSM 15229) were obtained from the DSMZ. *Rubrobacter xylanophilus* (DSMZ 9941) was a gift from Dr. Gaidamakova. *Rubrobacter *spp. were grown in TM medium containing 1 g/L tryptone, 1 g/L yeast extract, 0.7 g/L NaNO_3_, 0.1 g/L Na_2_HPO_4_, 0.1 g/L nitrilotriacetic acid, 0.1 g/L MgSO_4_·7H_2_O, 0.1 g/L KNO_3_, 60 mg/L CaSO_4_·2H_2_O, 8 mg/L NaCl, 2.2 mg/L MnSO_4_·H_2_O, 0.5 mg/L ZnSO_4_·7H_2_O, 0.5 mg/L H_3_BO_3_, 25 *μ*g/L CuSO_4_·5H2O,  25 *μ*g/L Na_2_MoO_4_·2H_2_O, 46 *μ*g/L CoCl_2_·6H_2_O, 10 ml/L 0.17 mM FeCl_3_·6H_2_O, final pH 8.2. Cultures were grown at 48°C for *R. radiotolerans *and at 60°C for *R. xylanophilus*, with shaking at 220 rpm in a Gyromax 737 shaker (Amerex Instruments, Lafayette, CA). *Pyrococcus furiosus* strain (DSMZ 3638) was grown in the absence of sulfur with 100 *μ*M Na_2_WO_4_ and 0.5% (wt/vol) maltose in the Pf medium containing 24 g/L NaCl, 4 g/L Na_2_SO_4_, 0.7 g/L KCl, 0.2 g/L NaHCO_3_, 0.1 g/L KBr, 0.03 g/L H_3_BO_3_, 10.8 g/L MgCl_2_·6H_2_O, 1.5/Lg CaCl_2_·2H_2_O, 0.025 g/L SrCl_2_·6H_2_O, 0.08% Na_2_S·9H_2_O, 5 g/L tryptone, 1 g/L yeast extract, 1 ml/L resazurin (0.2 g L^−1^ solution), final pH 6.8, in 100 mL serum bottles or 1 L bottles at 95°C under anaerobic conditions [46]. *Thermococcus gammatolerans *was grown in ASW-YTP medium containing 38 g/L NaCl, 14.5 g/L MgCl_2_·6H_2_O, 5 g/L tryptone, 5 g/L yeast extract, 5 g/L sodium pyruvate, 5.6 g/L MgSO_4_·7H_2_O, 2.5 g/L CaCl_2_·2H_2_O, 2.6 g/L Na_2_SO_4_, 1 g/L KCl, 80 mg/L Na_2_CO_3_, 80 mg/L NaBr, 64 mg/L KBr, 58 mg/L SrCl_2_·6H_2_O, 42 g/L H_3_BO_3_, 8.1 mg/L Na_2_HPO_4_, 2.4 mg/L NaF, 0.4 mg/L NaSiO_4_, 50 *μ*g/L KI, 0.08% Na_2_S·9H_2_O, 1 ml/L resazurin (0.2 g L^−1^ solution), final pH 6.8, in 100 mL serum bottles or 1 L bottles under anaerobic conditions at 88°C. 

### 2.2. Preparation of Enzyme-Free Cell Extracts

Cultures were grown in appropriate media and conditions to 0.4  OD_600 nm_  and the cells harvested by centrifugation at 8,000 ×g (10 min, 4°C). *Rubrobacter* spp. cells were washed twice with TM-BSS (TM media lacking tryptone and yeast extract, final pH 8.2), *P. furiosus* cells with Pf-BSS (Pf medium lacking carbon sources, tungsten, and Na_2_S·9H_2_O, final pH 6.8), and *T. gammatolerans* cells with ASW-BSS (ASW-YTP medium lacking carbon sources and Na_2_S·9H_2_O, final pH 6.8). Pellets were resuspended in distilled and deionized water (ddH_2_O, Sigma-Aldrich) and passed through an Emulsiflex Homogenizer (Avestin, Inc., Ottawa, Canada) at 15,000 psi to lyse the cells. Cell extracts were centrifuged at 12,000 ×g (60 min, 4°C) and standardized by protein concentration, which was determined by the BioRad Bradford Assay (BioRad, Hercules, CA). The supernatant was further centrifuged at 190,000 ×g (40 h, 4°C) and subjected to filtration using 3 kDa centrifugal devices (Amicon ultracel 3k filters; Millipore, Billerica, MA). The resulting protein-free cell extracts, called ultrafiltrates (UFs), were concentrated 5 times in a speed vacuum concentrator (Heto Vacuum Centrifuge; ATR, Laurel, MD) and stored at −20°C. The UF for *H. salinarum* was prepared as described in [11].

### 2.3. Enzyme Protection Assay

The restriction enzyme *Dde*I was added at a final concentration of 0.5 unit/*μ*L to UFs diluted to 0.2x, to 25 mM phosphate buffer (PiB), pH 7.0, to a 20 mM solution of trehalose, mannosylglycerate (MG), or di-*myo*-inositol phosphate (DIP), with or without the addition of 250 *μ*M or 25 *μ*M MnCl_2_. Assays performed under anaerobic conditions were purged with ultrahigh purity Ar (99.999%) (Valley National Gases, Frederick, MD). The solutions were irradiated on ice using a ^60^Co gamma source (Uniformed Services University of the Health Sciences, Bethesda, MD, dose rate 3.2 kGy/hr) at the following doses: 0, 1, 2, 3, 4, and 5 kGy or 0, 1, 2, 4, 6, 8, 10, and 12 kGy. Samples were kept on ice until digestion of 1 *μ*g of pUC19 DNA using 1 U of enzyme from each irradiated solution at 37°C for 1 h. The resulting pUC19 DNA fragments were separated by electrophoresis on 1% agarose TBE gels and visualized with ethidium bromide staining as previously described [11].

### 2.4. Determination of Amino Acid Concentration

Free and total amino acid concentrations in the UFs of *R. xylanophilus, R. radiotolerans, P. furiosus, *and *T. gammatolerans *were determined using the ninhydrin assay as previously described [11]. 

### 2.5. ICP-MS and Ion Chromatography

Mn, Fe, and PO_4_ concentration in *R. xylanophilus, R. radiotolerans, P. furiosus,* and* T. gammatolerans *UFs and cells (Mn, Fe) were determined using ICP-MS (Mn, Fe) and ion chromatography (PO_4_) at the Division of Environmental Health Engineering, JHU School of Public Health as previously described [11]. 

### 2.6. Preparation of Ethanol Extracts

Cells were harvested and washed with BSS. Pellets of 10^9^ cells were resuspended in 80% ethanol, broken via French press as previously described [47], and centrifuged at 10,000 ×g (50 min, 4°C). Cells and ethanol were on ice throughout the process.The ethanol was removed with a speed vacuum concentrator (Heto Vacuum Centrifuge, ATR, Laurel, MD), and the residue was resuspended in ultrapure water (ddH_2_O, Sigma-Aldrich) before filtration through a 10 kDa filter (Amicon ultracel 10k filters; Millipore, Billerca, MA). Cell protein concentration was determined by the BioRad Bradford Assay (Hercules, CA) using cell pellets of 10^9^ cells resuspended in distilled and deionized water (ddH_2_O, Sigma-Aldrich), lysed by French press, and centrifuged as described above. 

### 2.7. High-Performance Anion-Exchange Chromatography

High-performance anion-exchange chromatography (HPAEC) was carried out on Dionex DX 500 with a CarboPac PA-10 column and a PA-10 guard column (Dionex, Sunnyvale, CA) and pulsed amperometric detection (PAD). An aliquot of the ethanol extract was diluted 10- to 100-fold and injected into a CarboPac PA-1 column equilibrated with 16 mM sodium hydroxide. Elution was performed with a linear gradient from 16 mM sodium hydroxide to 0.5 M sodium acetate/0.1 M sodium hydroxide. Standards of 0.25, 0.5, 1, 2, and 4 nmol of trehalose, MG, and DIP were run for quantification. Mannosylglycerate (MG) and di-*myo*-inositol phosphate (DIP) were obtained from Bitop AG, Witten, Germany. 

## 3. Results

### 3.1. Composition Analysis of Ultrafiltrates

In previous studies, protein-free cell extracts, also called ultrafiltrates (UFs), of IR-resistant bacteria and archaea were found enriched in Mn^2+^ and small organic molecules that included amino acids and peptides [11, 15]. When combined* in vitro* at physiologically relevant concentrations, these constituents formed potent antioxidant complexes in orthophosphate buffer (PiB) [11, 15]. To determine the potential role of Mn and compatible solutes in the radiation resistance of thermophiles, we measured concentrations of metal ions, phosphates, and compatible solutes in whole cells and UFs of *R. xylanophilus, R. radiotolerans, P. furiosus, *and *T. gammatolerans* (Tables 1 and 2). UFs for the IR-resistant *Rubrobacter* species were enriched in Mn relative to that of IR-sensitive bacteria, yielding high Mn/Fe ratios similar to those found in *H. salinarum *(Table 1). The concentrations of Mn found in the UFs of the anaerobic archaea *T. gammatolerans* and *P. furiosus* were more than an order of magnitude lower than the values for the *Rubrobacter* species UFs, resulting in Mn/Fe ratios similar to that of the radiation-sensitive bacteria *E. coli* and *P. putida *(Table 1). The Mn/Fe ratios in whole cells followed the trend observed with the analysis of Mn/Fe ratios in the UFs (Table 1). Phosphate levels were high in all UFs with the exception of *P. furiosus* (Table 2).

We used high-performance anion-exchange chromatography (HPAEC) to quantify compatible solutes in UFs. *R. radiotolerans* and *R. xylanophilus *UFs both contained high amounts of trehalose with 29 mM and 17 mM, respectively. In addition, we found mannosylglycerate (MG) in UFs of both *R. xylanophilus *(99 mM) and *R. radiotolerans* (64 mM), whereas only the *R. xylanophilus* UF contained di-*myo*-inositol phosphate (DIP) (33 mM) (Table 2). *P. furiosus* UF had 52 mM of MG and 6 mM of DIP, which was significantly more than the concentrations found in the UF of *T. gammatolerans*. Amino acid and peptide concentrations were not significantly elevated in the *Rubrobacter* species UFs or that of *P. furiosus*, when compared with *H. salinarum* UF, whereas *T. gammatolerans* UF had a significantly higher free amino acid concentration (Table 2). Thus, the UFs of all the thermophiles reported here accumulated some small organic molecules but only the UFs of *R. radiotolerans* and *R. xylanophilus *exhibited significant amounts of Mn. 

To estimate intracellular concentrations of compatible solutes, we analyzed the ethanol extracts of our thermophilic organisms using HPAEC (Table 2). Our data for *P. furiosus* were similar to previously reported concentration of MG obtained by NMR, validating our methodology. Using cells grown in similar growth conditions with respect to salinity and temperature, we calculated an intracellular concentration of MG of 0.22 *μ*mol/mg protein versus 0.25 *μ*mol/mg protein reported by Martin and Santos [41]. We calculated the approximate intracellular concentration of MG and DIP for *P. furiosus* and *T. gammatolerans,* using a cellular volume of 4.5 *μ*L/mg protein [41]. In *P. furiosus*, MG and DIP concentrations were 49 mM and 10 mM, respectively, and in *T. gammatolerans*, we found concentrations of 21 mM for MG and 11 mM for DIP. These intracellular concentrations were similar to those for MG and DIP in the UFs of those organisms (Table 2). With regard to the *Rubrobacter *species, we did not have an appropriate cellular volume to calculate intracellular concentrations; however, Table 1 shows that both *R. radiotolerans *and* R. xylanophilus *had higher MG concentrations (and DIP concentrations for *R. xylanophilus*) than *P. furiosus* and *T. gammatolerans* in both the UFs and in our whole cells (ethanol extracts) determinations. 

To investigate the role of those small molecules in radiation resistance, we tested the ability of UFs and of reconstituted preparations, to protect the activity of purified enzymes exposed to increasing doses of IR.

### 3.2. Protection against IR by UFs and Compatible Solutes of Rubrobacter Species

**Figure 1:**
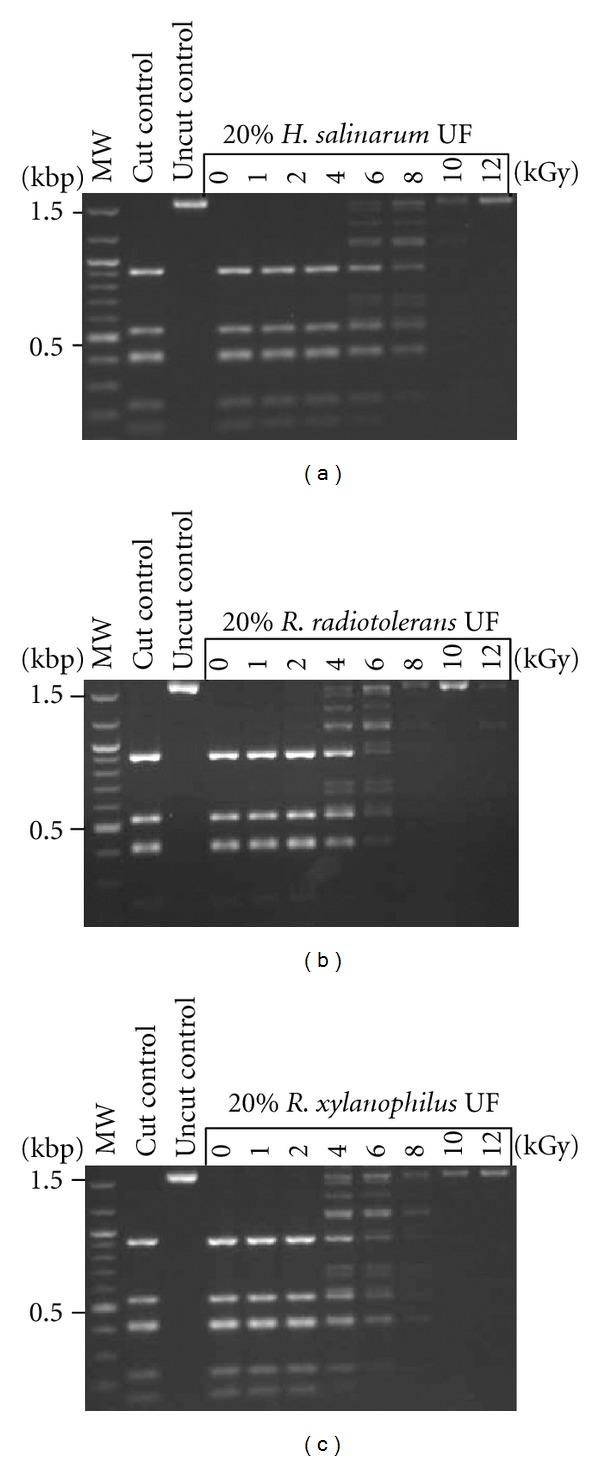
Protection of enzyme activity. The restriction enzyme *Dde*I was irradiated up to 12 kGy in enzyme-free cell extracts (UFs) of *H. salinarum*, *R. radiotolerans,* and *R. xylanophilus* (diluted to 0.2x). Residual restriction enzyme activity was assayed by the digestion of pUC19 plasmid DNA; fragments were analyzed by agarose gel electrophoresis. The first lanes are molecular size ladders.

**Figure 2:**
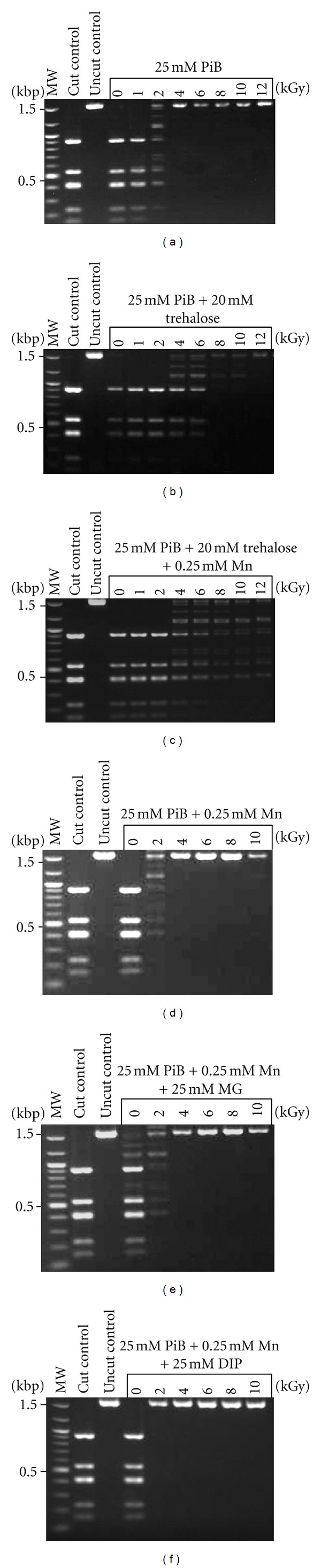
Protection of enzyme activity with compatible solutes under aerobic conditions. In upper panels, the restriction enzyme *Dde*I was irradiated up to 12 kGy with 25 mM phosphate buffer (PiB) and the addition of 20 mM trehalose and 0.25 mM Mn^2+^. In lower panels, the enzyme was irradiated up to 10 kGy, with 25 mM PiB combined with 0.25 mM Mn^2+^, with the addition of 25 mM MG or DIP. Residual restriction enzyme activity was assayed by the digestion of pUC19 plasmid DNA; fragments were analyzed by agarose gel electrophoresis. The first lanes are molecular size ladders.

We tested the radioprotective properties of UFs prepared from *R. xylanophilus *and *R. radiotolerans* on the activity of *Dde*I, a restriction endonuclease, exposed to doses of IR up to 12 kGy (Figure 1). Following irradiation, the residual activity of the enzyme was measured by its ability to cut plasmid DNA; the restriction fragments were analyzed by agarose gel electrophoresis. Under our experimental conditions, the *R. xylanophilus* and *R. radiotolerans *UFs provided protection of enzyme activity at doses extending to 6 and 8 kGy, respectively, which was comparable to levels of protection conferred by *H. salinarum* UF and significantly higher with the UF of IR-sensitive organisms (Figure 1; [11]). Next, we next tested the compatible solutes found in the UFs and the cells of both *Rubrobacter* species for their ability to protect enzyme activity against IR, at physiologically relevant concentrations. While the phosphate buffer (P_*i*_B) protected enzyme activity of 2 kGy, the addition of trehalose resulted in a significant increase in protection, up to 6 kGy (Figure 2). When trehalose and  PiB  were combined with 0.25 mM Mn^2+^ (determined to be physiologically relevant from the whole cell analysis), the radioprotection increased dramatically to 12 kGy. Irradiating the enzyme in PiB and Mn^2+^ alone only protected its activity to 2 kGy, and the addition of 25 mM MG or DIP did not increase protection (Figure 2).

### 3.3. Protection against IR by UFs and Compatible Solutes of Hyperthermophilic Archaea

**Figure 3:**
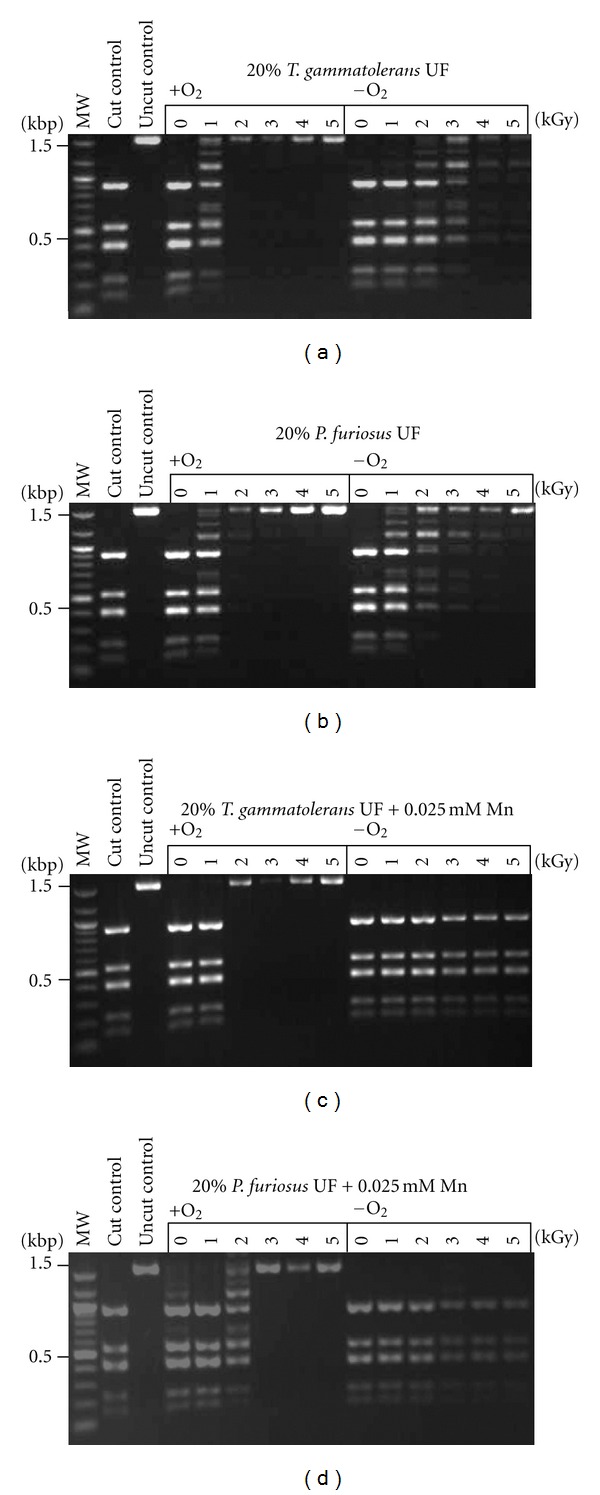
Protection of enzyme activity in aerobic and anaerobic conditions. The restriction enzyme *Dde*I was irradiated up to 5 kGy in the presence or absence of oxygen in enzyme-free cell extracts (UFs) of *T. gammatolerans *and *P. furiosus* (diluted to 0.2x). Residual restriction enzyme activity was assayed by the digestion of pUC19 plasmid DNA; fragments were analyzed by agarose gel electrophoresis. The first lanes are molecular size ladders.

In contrast to the *Rubrobacter* UFs, UFs of the anaerobes *P. furiosus* and *T. gammatolerans* did not protect *Dde*I activity at doses greater than 1 kGy under aerobic conditions (Figure 3). In these experiments, IR doses were increased with 1 kGy intervals to 5 kGy to increase resolution. To determine whether or not the lack of radioprotection was due to the presence of dioxygen (O_2_), we tested the UFs' properties under anaerobic conditions. In the absence of O_2_, UFs of *P. furiosus* and *T. gammatolerans* protected *Dde*I up to 3 kGy (Figure 3). The addition of 0.025 mM Mn^2+^ to UFs of *P. furiosus* and *T. gammatolerans *extended protection of the *Dde*I enzyme to 5 kGy, representing an increase of 2 kGy over aerobic conditions (Figure 3). While this Mn concentration (0.025 mM) was physiological relevant for *P. furiosus* and *T. gammatolerans*, it was 10- to 100-fold less than the Mn concentration found in the cells of the aerobic radiation resistant *Rubrobacter* species (Table 1). 

**Figure 4:**
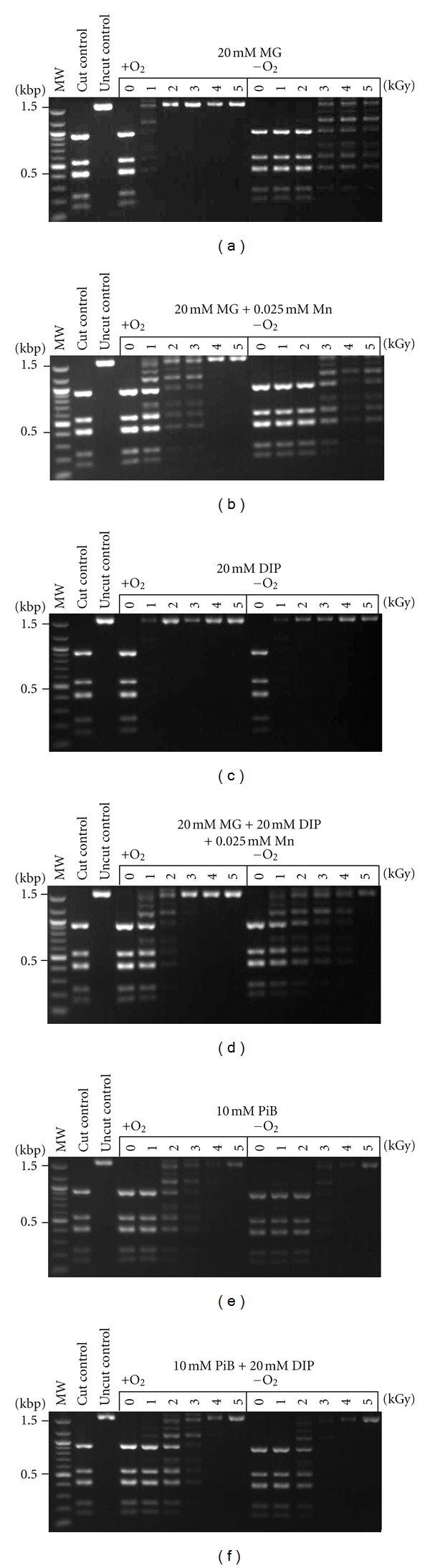
Protection of enzyme activity with compatible solutes. The restriction enzyme *Dde*I was irradiated up to 5 kGy in the presence or absence of oxygen, in water or with the addition of 20 mM MG, 20 mM MG and 0.025 mM Mn, 20 mM DIP, or 20 mM MG, 20 mM DIP, and 0.025 mM Mn. The 20 mM solution of DIP had a pH of 9.5; thus 10 mM  PiB was added for a final pH of 7.5. Residual restriction enzyme activity was assayed by the digestion of pUC19 plasmid DNA; fragments were analyzed by agarose gel electrophoresis. The first lanes are molecular size ladders.

We also compared the enzyme protection activity of MG and DIP in the presence and absence of O_2_. Under the anaerobic conditions found in the intracellular milieu of *P. furiosus* and *T. gammatolerans*, MG protection of the *Dde*I enzyme was extended to 5 kGy, from only 1 kGy under aerobic conditions (Figure 4). Protection of enzyme activity was also extended under both aerobic and anaerobic conditions when the enzyme was irradiated with MG and Mn^2+^. DIP did not show any increase in enzyme protection, either alone or in combination with MG and Mn^2+^, but rather caused a decrease in enzyme protection. In fact, the level of protection afforded by PiB alone was identical to that with PiB and 20 mM DIP. We also found that PiB was more protective than 20 mM of MG alone. These experiments show that incubation of the enzyme under anaerobic conditions during irradiation was the single most effective condition for extending enzyme activity to higher doses of IR. 

## 4. Discussion 

Thermophiles are defined by their requirement of high temperatures for growth, but within that group there is a great diversity of metabolic capabilities and of environments inhabited by these microorganisms [48]. Here we investigated the radiation resistance of two groups of thermophiles that are phylogenetically and metabolically distinct. The bacteria, *R. xylanophilus *and *R. radiotolerans,* are IR-resistant thermophiles from aerobic environments and the archaea, *T. gammatolerans *and *P. furiosus,* are IR-resistant hyperthermophiles from anaerobic environments. 

A tight correlation between high radiation resistance in bacteria and archaea and high intracellular Mn/Fe ratios has been established from studies with model organisms and environmental isolates [11, 22, 49]. Both *R. xylanophilus* and *R. radiotolerans* exhibited Mn/Fe ratio similar to that of the IR-resistant *D. radiodurans* and *H. salinarum*, emphasizing the central role played by Mn in the radiation resistance of prokaryotes [7]. Previous studies showed that Mn^2+^ boosts protein protection in cells by interacting synergistically with the pool of small molecules, including orthophosphate, amino acids, peptides, and nucleosides, generating catalytic O_2_^•−^- and H_2_O_2_-scavenging complexes [11, 50, 51]. Both *Rubrobacter* species' UFs were enriched in Mn and phosphate and protected enzyme activity, *in vitro*, from similarly high doses of IR compared to the UF from *H. salinarum *[11]. These findings suggested that Mn-associated antioxidant molecules might also be present in the *Rubrobacter* spp., providing *in vivo* protection to all the cell's macromolecules by mitigating the effect of IR-generated ROS [11, 50]. The *Rubrobacter* UFs did not contain high concentrations of amino acids or peptide, as was found in *D. radiodurans* and *H. salinarum* but they were enriched in compatible solutes that included trehalose, MG, and DIP [11, 15, 17].

Compatible solutes of thermophiles have been studied extensively for their protein-stabilizing properties [44]. A number of thermophiles are radiation-resistant, and herein we evaluated the possible antioxidant properties of these molecules with respect to radiation. Prior to this work, the compatible solutes present in *R. radiotolerans *had not been investigated. We found that this bacterium accumulated trehalose and MG to similar amounts as *R. xylanophilus*, but not DIP when grown at 48°C. DIP is associated mostly with hyperthermophiles (optimal growth temperature >80°C), and *R. radiotolerans *is considered moderately thermophilic with an optimal growth temperature of 48°C [52]. *R. xylanophilus*, in contrast, has an optimal growth temperature of 60°C, which is the lowest growth temperature reported among organisms known to accumulate DIP [53]. As previously described, *R. xylanophilus *accumulates trehalose, MG, and DIP under its optimal growth conditions and increases the concentration of these compatible solutes in response to heat or osmotic stress [37]. We have determined that *R. radiotolerans *also maintains basal millimolar cellular concentrations of trehalose and MG, representing organic solutes constitutively present in the cell, with potential for antioxidant properties. While it was unexpected that MG and DIP did not protect enzyme activity *in vitro*, these compatible solutes carry negative charges and might be repelled at various negatively charged sites on a protein, leaving areas susceptible to ROS attacks. Although, both compounds were previously shown to scavenge HO^•^ [44], we conclude here that they are not efficient scavengers of O_2_^•−^ and H_2_O_2_, generated by exposure to IR [1].

Trehalose, a compatible solute of similar size as MG and DIP but carrying no charges, was highly protective of protein activity against IR, either alone or in combination with Mn^2+^. Trehalose is present in a wide variety of organisms including bacteria, yeast, fungi, plants, and invertebrates and was found to protect proteins from heat, osmotic stress, desiccation, and oxidation [54]. Additionally, strains of *Chroococcidiopsis*, a desiccation and IR-resistant cyanobacterium, were shown to accumulate trehalose [55, 56]. Here we demonstrated experimentally the antioxidant properties of trehalose and proposed that, in combination with Mn and phosphate, this small organic molecule forms the basis for the high radiation resistance found in *R. xylanophilus *and* R. radiotolerans*. These findings fit the current model of Mn-based antioxidants scavenging IR-generated ROS that was established for aerobic bacteria and archaea [7, 11, 51]. In addition to its antioxidant activity, Mn may also act by functionally substituting for Fe in the Fe–S cluster of enzymes and thereby mitigating the deleterious effects of Fenton chemistry during oxidative stress [57].

The basis for the radiation resistance of the anaerobic hyperthermophiles *P. furiosus* and *T. gammatolerans* seems to be quite different from that of the aerobic thermophiles. Both organisms exhibited low Mn/Fe ratios that were similar to those of radiation-sensitive *E. coli* and *P. putida* [9]. This is in conflict with the model of Mn^2+^-dependent ROS scavenging for aerobic bacteria and archaea [7, 11, 51]. However, a number of proteins in anaerobes require Fe such as dehydrogenases and ferredoxin, an electron carrier that *P. furiosus *uses in place of NAD [58–60]. *P. furiosus*, like most anaerobic hyperthermophiles, lacks the oxygen detoxification enzymes superoxide dismutase (SOD) and catalase that are used by their aerobic counterparts [61]. Instead, *P. furiosus* has a superoxide reductase (SOR), a nonheme iron-containing enzyme that catalyzes the reduction of O_2_^•−^ into H_2_O_2_, and several peroxidases, including rubrerythrin, and alkyl hydroperoxide reductase I and II [61, 62]. Furthermore, while Mn^2+^-dependent ROS scavenging of O_2_^•−^ and H_2_O_2_—which are formed predominantly under aerobic irradiation—is essential in the presence of oxygen, it might not be the case under anaerobic conditions [63, 64]. In the presence of O_2_, the formation of O_2_^•−^ is a 1-step process in which a free electron (e^−^) reacts with O_2_ (2.0 10^10^ M^−1^ s^−1^). This is significantly faster than in the absence of O_2_ where the formation of O_2_^•−^ is dependent upon concentrations of HO^•^ and H_2_O_2_ (2.7 10^7^ M^−1^ s^−1^) [63]. In our *in vitro* protection assay, *P. furiosus* and *T. gammatolerans *UFs displayed increased protection under anaerobic conditions, suggesting that one element of their radiation resistance might be attributed to the anaerobic environment itself. Another element is likely to be their metabolic adaptations to maintaining strict anaerobic conditions in their intracellular milieu. 

While ROS detoxification enzymes were shown to be dispensable for the survival of the aerobic archaeon *H. salinarum* to IR [11], a whole-genome mRNA microarray analysis of *P. furiosus* in response to radiation showed that genes encoding for a putative Dps-like iron-chelating protein and two membrane-bound oxidoreductases were differentially expressed following IR, potentially in response to oxidative stress [65]. The most interesting finding was the high-level constitutive expression of many systems involved in oxygen detoxification and redox homeostasis, presumably to protect cellular proteins from oxidative damage [65]. Similarly, genes in the SOR pathway were most highly expressed in *P. furiosus* under normal anaerobic growth conditions, and no increased expression of SOR was found in response to IR or H_2_O_2_, indicating that this protein may be functioning at maximum capacity at all times [59, 62, 65]. A variation of SOR-mediated O_2_^•−^ detoxification was recently discovered in *Desulfoarculus baarsii* in which SOR complexed with ferrocyanide reduces O_2_^•−^ without the formation of H_2_O_2_. This system is highly efficient, as the SOR iron site remains reduced, thus eliminating the requirement of oxidoreductases to recycle SOR [66]. We propose that low levels of IR-generated ROS under anaerobic conditions combined with highly constitutively expressed detoxification systems in the anaerobic hyperthermophiles, *P. furiosus* and *T. gammatolerans*, are key to their radiation resistance and circumvent the need for the accumulation of Mn-antioxidant complexes in the cell.

The study of extremophiles and how they meet the physical and chemical challenges found in the environmental extremes they inhabit has led to many new insights into the mechanisms of stress response. Previous work, together with the mechanisms underlying the radiation resistance of the thermophiles described here, underscores the multiple strategies microorganisms can use to escape environmental stresses. The variety of Mn-antioxidant complexes found so far suggests that the adaptations of extremophiles to their environments provide a tremendous reservoir for novel radioprotective molecules and antioxidants against the deleterious effects of IR. One question remains: is Mn a universal feature of IR resistance in aerobic systems, and does the model of Mn^2+^-dependent ROS scavenging extend to eukaryotic systems? 

## Figures and Tables

**Table 1:** Concentrations of Mn and Fe in ultrafiltrates (UFs) and whole cells of thermophiles and radiation-sensitive bacteria.

		Conc. In:						
			Ultrafiltrates			Whole cells		
Organism	*D*_10_^a^	Genome	Mn	Fe	Mn/Fe	Mn	Fe	Mn/Fe
	(kG)	(Mbp)	(*μ*M)	(*μ*M)		(ng/10^9^ cells)	(ng/10^9^ cells)	
*P. putida* ^ b^	0.1	6.2	0.9	6.1	0.1	18	1045	0.02
*E. coli* ^ b^	0.5	4.6	0.6	3.5	0.2	14	645	0.02
*H. salinarum* ^ b^	5	2.6	87	8.9	9.8	155	818	0.19
*R. xylanophilus *	6	3.2	79	8.2	9.6	549	290	1.9
*R. radiotolerans *	10	3.4	211	18	11.8	300	340	0.88
*P. furiosus *	3	1.9	5.3	113	0.1	14	345	0.04
*T. gammatolerans *	6	2.1	6.3	15	0.4	3	235	0.01

^a^Dose at which viable cells are reduced to 10% of the population.

^b^From [11].

**Table 2:** Concentrations of amino acids, PO_4_, and compatible solutes in thermophiles and radiation-sensitive bacteria UFs and thermophiles ethanol extracts.

	Conc. In:								
	Ultrafiltrates (mM)						Ethanol extracts (*μ*mol/mg protein)		
Organism	Amino Acids		PO_4_	Trehalose	MG	DIP	Trehalose	MG	DIP
	Free	Total							
*P. putida* ^ a^	52	121	4.5	nd	nd	nd			
*E. coli* ^ a^	80	181	5.9	nd	nd	nd			
*H. salinarum* ^ a^	325	642	22	nd	nd	nd			
*R. xylanophilus *	87	115	10	17	99	33	1.5	3.0	0.7
*R. radiotolerans *	134	159	24	29	64	—^b^	1.7	2.4	nd
*P. furiosus *	15	35	5.4	nd	52	6	nd	0.2	0.04
*T. gammatolerans *	221	235	19	nd	10	2.3	nd	0.1	0.05

^a^From [11].

^b^Not detected.

nd: not determined.
