# Erratum to “Role of Mn^**2+**^ and Compatible Solutes in the Radiation Resistance of Thermophilic Bacteria and Archaea”

**DOI:** 10.1155/2013/810286

**Published:** 2013-01-10

**Authors:** Kimberly M. Webb, Jocelyne DiRuggiero

**Affiliations:** Department of Biology, Johns Hopkins University, Baltimore, MD 21218, USA

After our paper was published online, we found an error in the last paragraph of the introduction. The word “mitigated” in the sentence “We showed that under aerobic conditions, compatible solutes accumulated by thermophilic bacteria confer IR resistance to enzymes *in vitro *and that radioprotection is mitigated by the presence of both trehalose and Mn^2+^” should be correctly written as “mediated.”

